# Supplementary material for: Causal Relationship between Aging and Anorexia Nervosa: A White-Matter-Microstructure-Mediated Mendelian Randomization Analysis
Source: Biomedicines. 2024 Aug 16;12(8):1874. doi: 10.3390/biomedicines12081874 (PMC11351342; doi:10.3390/biomedicines12081874)
Supplement: Supplementary file 1 [file biomedicines-12-01874-s001.zip › Supplementary Figure/Figure S4/Singe SNP plot of MR analysis for TL to ubm-b-2077.pdf]

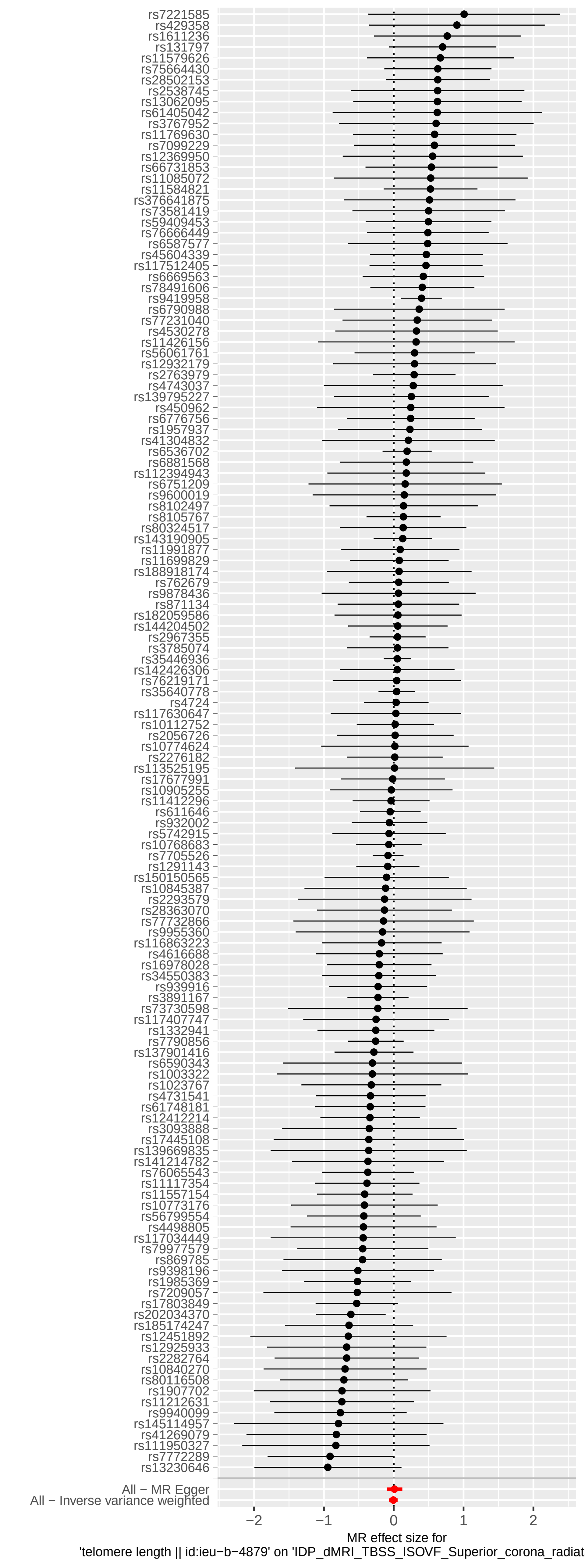
rs7221585
rs429358
rs1611236
rs131797
rs11579626
rs75664430
rs28502153
rs2538745
rs13062095
rs61405042
rs3767952
rs11769630
rs7099229
rs12369950
rs66731853
rs11085072
rs11584821
rs376641875
rs73581419
rs59409453
rs76666449
rs6587577
rs45604339
rs117512405
rs6669563
rs78491606
rs9419958
rs6790988
rs77231040
rs4530278
rs11426156
rs56061761
rs12932179
rs2763979
rs4743037
rs139795227
rs450962
rs6776756
rs1957937
rs41304832
rs6536702
rs6881568
rs112394943
rs6751209
rs9600019
rs8102497
rs8105767
rs80324517
rs143190905
rs11991877
rs11699829
rs188918174
rs762679
rs9878436
rs871134
rs182059586
rs144204502
rs2967355
rs3785074
rs35446936
rs142426306
rs76219171
rs35640778
rs4724
rs117630647
rs10112752
rs2056726
rs10774624
rs2276182
rs113525195
rs17677991
rs10905255
rs11412296
rs611646
rs932002
rs5742915
rs10768683
rs7705526
rs1291143
rs150150565
rs10845387
rs2293579
rs28363070
rs77732866
rs9955360
rs116863223
rs4616688
rs16978028
rs34550383
rs939916
rs3891167
rs73730598
rs117407747
rs1332941
rs7790856
rs137901416
rs6590343
rs1003322
rs1023767
rs4731541
rs61748181
rs12412214
rs3093888
rs17445108
rs139669835
rs141214782
rs76065543
rs11117354
rs11557154
rs10773176
rs56799554
rs4498805
rs117034449
rs79977579
rs869785
rs9398196
rs1985369
rs7209057
rs17803849
rs202034370
rs185174247
rs12451892
rs12925933
rs2282764
rs10840270
rs80116508
rs1907702
rs11212631
rs9940099
rs145114957
rs41269079
rs111950327
rs7772289
rs13230646
All − MR Egger
All − Inverse variance weighted
−2
−1
0
1
2
MR effect size for
'telomere length || id:ieu−b−4879' on 'IDP_dMRI_TBSS_ISOVF_Superior_corona_radiat
